# A hybrid reanalysis-forecast meteorological forcing data for advancing climate adaptation in agriculture

**DOI:** 10.1038/s41597-024-03702-5

**Published:** 2024-08-08

**Authors:** Toshichika Iizumi, Takahiro Takimoto, Yoshimitsu Masaki, Atsushi Maruyama, Nobuyuki Kayaba, Yuhei Takaya, Yuji Masutomi

**Affiliations:** 1 Institute for Agro-Environmental Sciences, National Agriculture and Food Research Organization, 3-1-3 Kannondai, Tsukuba, Ibaraki 305-8604 Japan; 2 Japan Meteorological Agency, 3-6-9 Toranomon, Minato City, Tokyo 105-8431 Japan; 3 Meteorological Research Institute, 1-1 Nagamine, Tsukuba, Ibaraki 305-0052 Japan; 4 Center for Climate Change Adaptation, National Institute for Environmental Studies, 16-2 Onogawa, Tsukuba, Ibaraki 305-8506 Japan

**Keywords:** Climate-change impacts, Agriculture

## Abstract

Climate variability in the growing season is well suited for testing adaptation measures. Adaptation to adverse events, such as heatwaves and droughts, increases the capacity of players in agri-food systems, not only producers but also transporters and food manufacturers, to prepare for production disruptions due to seasonal extremes and climate change. Climate impact models (e.g., crop models) can be used to develop adaptation responses. To run these models, historical records and climate forecasts need to be combined as a single daily time series. We introduce the daily 0.5° global hybrid reanalysis-forecast meteorological forcing dataset from 2010 to 2021. The dataset consists of the Japanese 55-yr Reanalysis (JRA55) and the Japan Meteorological Agency/Meteorological Research Institute Coupled Prediction System version 2 (JMA/MRI-CPS2) 5-member ensemble forecast. Both are bias-corrected using the Delta method and integrated with a baseline climatology derived from the Environmental Research and Technology Development Fund’s Strategic Research 14 Meteorological Forcing Dataset (S14FD). The dataset is called JCDS (JRA55-CPS2-Delta-S14FD) and offers a framework for monitoring and forecasting applications towards adaptation.

## Background & Summary

Year-to-year variations in climatic conditions during the growing season, especially episodes with marked detrimental effects on crop growth, pose a considerable risk to agriculture^1–5^. Producers must respond to fluctuations in pre-sowing soil moisture conditions, water availability, and crop growth, and adjust their management practices when feasible. Known practices include shifting planting dates, switching varieties or crop species, and modifying the timing and amount of water, fertilizer, and chemical inputs^6,7^. These adjustments are important to ensure that the harvested crops meet the quality standards required by markets and consumers^8,9^. The knowledge and experience of producers, combined with information from agricultural extension services and decision support systems, all help to mitigate the negative effects of unfavorable climate conditions through agronomic adjustments^7,10,11^.

Some adaptations to climate change are the result of producers’ responses to natural climate variability^12^. If producers can develop adaptation measures capable of mitigating the negative impacts of extremes in the current climate (e.g., heatwaves, drought, and heavy rainfalls associated with major modes of climate variability, such as El Niño-Southern Oscillation), then it is possible that some of these measures might be well suited for sustaining agricultural activities under a future climate^13^. Similarly, the development and implementation of adaption measures by other players in agri-food systems, including policymakers in charge of trade agreements, food traders, millers, and logistic businesses, could also mitigate the negative effects of climate change^14^. Consequently, enhancing players’ capacity to cope with seasonal climate-induced production shortfalls through seasonal climate and crop forecasts would improve their adaptive capacity and preparedness for climate change^15,16^, and is increasingly important as the food supply chains have become globally interconnected.

To derive possible early response scenarios to climate-induced production shortfalls and associated market disruptions, analysts need to employ climate impact models (e.g., crop models and agro-economic models) with meteorological forcing data that combine historical records and seasonal climate forecasts^17,18^. However, such hybrid reanalysis-forecast datasets are often developed for specific research projects and development is often not continued after that research project ends. Indeed, only some datasets are provided on a continuous basis. For example, the Monitoring of Agriculture with Remote Sensing (MARS) yield forecasting system that is operated by the Joint Research Centre of the European Commission uses the European Centre for Medium-Range Weather Forecasts (ECMWF) 10-day forecasts to run the WOrld FOod STudies (WOFOST) crop model^19^. However, the generated data are for internal use only and are not open to the public. Another example of such hybrid datasets is the Agro-Meteorological Grid Square Data which are provided continually by the National Agriculture and Food Research Organization (NARO). The data comprise a hybrid of weather observations and the Japan Meteorological Agency (JMA) 26-day forecasts for agricultural decision support in Japan^20^. However, no global datasets of this kind have been made publicly available to date, making it difficult to benchmark the skill of crop forecasts in many regions of the world when comparing multiple crop forecast methods using climate forecasts from different centers as the inputs.

To address this issue, we developed a daily 0.5° global bias-corrected hybrid reanalysis-forecast dataset, which we refer to as JCDS. The JCDS dataset described here is a hybrid of the JRA55 reanalysis data and JMA/MRI-CPS2 forecast data from 2010 to 2021. The JCDS dataset contains 10 variables that are widely used to run climate impact models (Table 1). The dataset described here includes forecasts for historical periods, which enables users to assess the predictive performance of a specific application when the JCDS data are used as the input. Moreover, in the Usage Notes, we show how the JCDS data can be used with a global gridded crop model (GGCM) to produce large-scale crop forecasts for the world’s major exporting country, providing food-importing countries with information to prepare proactive responses. The method harnesses early responses of players in agri-food systems to seasonal climate-induced production shortfalls and, ultimately, helps to develop adaptation measures. As a test case, we used the rice production shortfall caused by the drought that hit Southeast Asia in 2015 and 2016.

**Table 1:** Climate variables available in the JCDS dataset.

Symbol	Unit	Description
*T*_ave_	°C	Daily mean 2-m air temperature
*T*_max_	°C	Daily maximum 2-m air temperature
*T*_min_	°C	Daily minimum 2-m air temperature
*P*	mm d^–1^	Precipitation rate
*SW*	W m^–2^	Downward shortwave radiation flux
*LW*	W m^–2^	Downward longwave radiation flux
*RH*	%	2-m relative humidity
*Q*	kg kg^–1^	2-m specific humidity
*W*	m s^–1^	10-m wind speed
*P*_s_	hPa	Surface pressure

## Methods

**Fig. 1:**
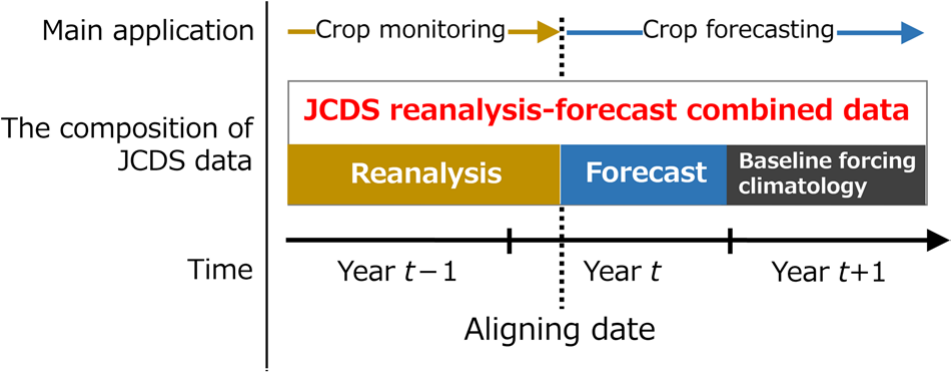
Composition of JCDS data for a given alignment date and its main application.

The JCDS data consist of global atmospheric reanalysis, dynamic seasonal climate forecast, and a climatology derived from baseline meteorological forcing data (Fig. 1). These components were integrated into a daily time series following bias-correction. We employed the JRA55 reanalysis data^21^ and the JMA/MRI-CPS2 5-member ensemble 241-day forecast^22^, issued twice per month (i.e., yielding 24 initial conditions annually). In addition, we utilized the S14FD retrospective meteorological forcing data^23^ and the delta bias-correction method^24,25^. The resulting hybrid dataset is designated as JRA55-CPS2-Delta-S14FD, which we abbreviated to JCDS. The JRA55 data (http://search.diasjp.net/en/dataset/JRA55) and S14FD data^26^ were obtained from the Data Integration and Analysis System (DIAS), and the JMA/MRI-CPS2 data were purchased from the Japan Meteorological Business Support Center (http://www.jmbsc.or.jp/jp/online/file/f-online10650.html). The subsequent subsections describe the methods used to process the reanalysis, forecast, and forcing data. Processing included spatial interpolation, elevation adjustment, non-output variable estimation, and unit conversion, as well as bias-correction and integration with baseline climatology.

### Reanalysis

First, we spatially interpolated 3-hourly data for 10 variables in the 2-dimensional instantaneous and average diagnostic fields (Table 2) from an original resolution of 0.5625° to a 0.5° grid using the inverse distance weighting method. The interpolated east-west and south-north components of the 10-m wind speed were merged into a single scalar wind speed (*W*). Daily mean, maximum, and minimum values of 2-m air temperature (*T*_ave_, *T*_max_, and *T*_min_), as well as the diurnal temperature range (*DTR*), were calculated from eight 3-hourly data points (0 UTC to 21 UTC). The units of these temperature variables were converted from K to °C. Daily accumulated values were calculated for precipitation rate (*P*), whereas daily means were calculated for the remaining variables. The unit of surface pressure (*P*_s_) was converted from Pa to hPa. No adjustments were made for elevation as the difference in mean elevation before and after spatial interpolation was minor, and any biases due to elevation discrepancies were removed in the bias-correction. After completing these processing steps for the 1981–2010 period, daily climatological mean values were calculated and used in bias correction.

**Table 2:** Reanalysis variables and their correspondence with JCDS variables.

JRA55 reanalysis variables				JCDS variables (daily/0.5°)
Output category	Variable	Unit	Description	
Two-dimensional instantaneous diagnostic fields (fcst_surf; 3-hourly/0.5625°)	tmp2m	K	2-m air temperature	*T*_ave,_ *T*_max,_ *T*_min_
	rh2m	%	2-m relative humidity	*RH*
	spfh2m	kg kg^–1^	2-m specific humidity	*Q*
	ugrd10m	m s^–1^	10-m east-west wind speed	*W*
	vgrd10m	m s^–1^	10-m south-north wind speed	
	tcdc	%	90–1100 hPa total cloud cover	
Two-dimensional average diagnostic fields (fcst_phy2m; 3-hourly/0.5625°)	tpratsfc	mm d^–1^	Precipitation rate	*P*
	dswrfsfc	W m^–2^	Downward shortwave radiation flux	*SW*
	dlwrfsfc	W m^–2^	Downward longwave radiation flux	*LW*
	pressfc	Pa	Surface pressure	*P*_s_
Isobaric analysis fields (anl_p125; 6-hourly/1.25°)	rhp850	%	850-hPa relative humidity	
	ugrdp850	m s^–1^	850-hPa east-west wind speed	
	vgrdp850	m s^–1^	850-hPa south-north wind speed	

In addition, we acquired the 0.5625° 3-hourly total cloud cover from 90-hPa to 1,100-hPa levels (*C*), as well as the 1.125° 6-hourly relative humidity and east-west and south-north components of wind speed at the 850-hPa level (*RH*_p850_, *U*_p850_, and *V*_p850_) (Table 2). After spatial interpolation, the *U*_p850_ and *V*_p850_ components were merged (*W*_p850_). Daily mean values of *C*, *RH*_p850_, and *W*_p850_ were calculated and used to estimate the non-output variables in the forecasting model.

### Forecasting

Daily data of seven variables at a 2.5° resolution (Table 3) were interpolated to a 0.5° resolution. Subsequently, the east-west and south-north components of wind speed at the 850-hPa level were merged. Adjustment for elevation, estimation of non-output variables, and unit conversions were performed as described below. These processes were performed for each of the 24 initial conditions and five ensemble members. Finally, daily climatological mean values for the 1981–2010 period were then calculated by averaging across the initial conditions and ensemble members. Seasonal climate forecasts for the subsequent 241 days from each initial date were generated using slightly different initial conditions, which are represented by the different ensemble members. The differences among ensemble members give users an indication of the likelihood that a particular seasonal climate state and its impact will be above, near, or below normal.

**Table 3:** Forecast variables and their correspondence with JCDS variables.

JMA/MRI-CPS2 forecast variables (daily/2.5°)				JCDS variables (daily/0.5°)
Output category	Variable	Unit	Description	
Surface	h2_Ptt	K	2-m air temperature	*T*_ave,_ *T*_max,_ *T*_min_
	surf_Prr	mm d^–1^	Precipitation rate	*P*
	surf_Ppp	Pa	Sea level pressure	*P*_s_
850-hPa level	p850_Ptt p850_Prh	K %	850-hPa air temperature 850-hPa relative humidity	*RH*
				*Q*
				*SW*
				*LW*
	p850_Pwu	m s^–1^	850-hPa east-west wind speed	*W*
	p850_Pwv	m s^–1^	850-hPa south-north wind speed	

#### Temperature

Daily mean 2-m air temperature was adjusted to account for the mean elevation difference between the 2.5° and 0.5° grid cells using the environmental lapse rate (*γ* = 0.0065 K m^–1^). Daily maximum and minimum temperatures were then derived based on the climatological mean *DTR* from the reanalysis data.

#### Surface pressure

Sea level pressure data were converted to the surface pressure values that corresponded to the mean elevation at the 0.5° grid resolution as follows:

1 $${P}_{{\rm{s}}}={P}_{0}{\left(\frac{{T}_{0}-\gamma z}{{T}_{0}}\right)}^{g/{\gamma R}_{{\rm{a}}}}$$

where *P*_*s*_ is the surface pressure (Pa), *P*_0_ is the sea level pressure (Pa), *z* is the mean elevation (m), *T*_0_ is the daily mean air temperature at sea level (°C) and calculated from 2-m air temperature, *g* is the gravitational acceleration (9.81 m s^−2^), and *R*_a_ is the gas constant of air (287 J kg^−1^ K^−1^). After the elevation adjustment, the unit of surface pressure was converted to hPa.

#### Relative and specific humidity

Vapor pressure at the 850-hPa level was derived from relative humidity and temperature at the same level (p850_Ptt and p850_Prh; Table 3). Then, relative humidity at 2-m height was calculated using the aforementioned vapor pressure and the 2-m air temperature (h2_Ptt) by assuming that the vapor pressure values remained constant between the 850-hPa and surface levels. Then, the 2-m specific humidity, *Q* (kg kg^–1^), was derived as follows:

2 $$Q=\frac{0.622e}{{P}_{{\rm{s}}}-0.378e}$$

where *e* is the vapor pressure (hPa) and *P*_s_ is the surface pressure (hPa).

#### Downward shortwave and longwave radiation fluxes

Since no radiation output was available in the forecast, downward shortwave and longwave radiation fluxes were estimated empirically. According to previous studies^27,28^, relationships were initially established using the reanalysis data:

3 $$\mathrm{ln}\left(C\right)={a}_{0}+{a}_{1}\mathrm{ln}\left({{RH}}_{{\rm{p}}850}\right)$$

where *C* is the total cloud cover from the 90 hPa to 1,100 hPa levels (%), *RH*_p850_ is the 850-hPa relative humidity (%), and *a*_0_ and *a*_1_ are regression coefficients. These coefficient values were determined for each 0.5° grid cell and day of the year using daily data for the 1981–2010 period with an 11-day centered moving window (n=330). Then, based on the known relationships between cloud cover and downward radiations^29^, the following relationships were formulated:

4 $${SW}={b}_{0}+{b}_{1}\mathrm{ln}(C)\,{\rm{and}}\,{LW}={c}_{0}+{c}_{1}\mathrm{ln}(C)$$

where *SW* and *LW* are downward shortwave and longwave radiation fluxes (W m^−2^), respectively, and *b*_0_, *b*_1_, *c*_0_, and *c*_1_ are the regression coefficients. These coefficient values were determined in the same manner as that used to establish the relationships between cloud cover and relative humidity.

#### Wind speed

Wind speeds at the 10-m height were estimated from those at the 850-hPa level. Based on the findings of Torralba *et al*.^30^, the correlations between the wind speeds at the two levels were established for each grid cell and day of the year using the reanalysis data as follows:

5 $$W={d}_{0}+{d}_{1}{W}_{{\rm{p}}850}$$

where *W* and *W*_p850_ are wind speed at 10-m and 850-hPa levels, respectively; *d*_0_ and *d*_1_ are the regression coefficients determined in the same manner as described above. The logarithmic profile commonly employed to describe wind speeds in the surface layer of the atmospheric boundary layer cannot be applied to the 850-hPa level, which is situated at approximately 1,500 m in the standard atmosphere.

#### Precipitation rate

Unlike other variables, we did not perform any preprocessing, such as elevation adjustment, for precipitation rate.

**Baseline forcing climatology**. Daily averages for the period 1981–2010 were calculated using S14FD for the terrestrial areas and JRA55 for Antarctic and ocean areas, establishing the baseline forcing climatology. In addition, daily minimum non-zero precipitation rate values were calculated for each grid cell and day of the year and employed as thresholds (*P*_tr_) in the bias-correction process to moderate the influence of excessive many wet days with minimal precipitation.

**Bias-correction**. The delta method^24,25^ was used to correct for biases in the processed daily reanalysis and forecast data. For the temperature variables (*T*_ave_, *T*_max_, *T*_min_), the additive form was applied as follows:

6 $${x}_{y,m,d}^{{\rm{bc}}}={\bar{x}}_{m,d}^{{\rm{base}}}+{x}_{y,m,d}^{{\rm{reanl}}}-{\bar{x}}_{m,d}^{{\rm{reanl}}}$$

where the subscripts *y*, *m*, and *d* are the year, month, and day, respectively. ${x}^{{\rm{bc}}}$ is the bias-corrected value, ${x}^{{\rm{reanl}}}$ is the reanalysis value, and ${\bar{x}}^{{\rm{base}}}$ and ${\bar{x}}^{{\rm{reanal}}}$ are the climatological mean value for the 1981–2010 period calculated from the baseline forcing data and the reanalysis, respectively. The additive form was also used for *P*_s_ and *W*, whereas the multiplicative form was utilized for the other variables (*RH*, *Q*, *SW*, *LW*, and *P*):

7 $${x}_{y,\,m,d}^{{\rm{bc}}}=\frac{{x}_{y,m,d}^{{\rm{reanl}}}}{{\bar{x}}_{m,d}^{{\rm{reanl}}}}\times {\bar{x}}_{m,d}^{{\rm{base}}}$$

After the bias correction, variable-specific lower and upper bounds were imposed on the bias-corrected values when necessary (0≤*RH*≤100, 0≤*Q*, 0≤*SW*, 0≤*LW*). Precipitation values falling below the threshold *P*_tr_ were replaced with zero. For the bias-correction of daily forecast data (${x}^{{\rm{forecast}}}$), the forecast climatological mean ${\bar{x}}^{{\rm{forecast}}}$ was used instead of ${\bar{x}}^{{\rm{reanal}}}$.

**Fig. 2:**
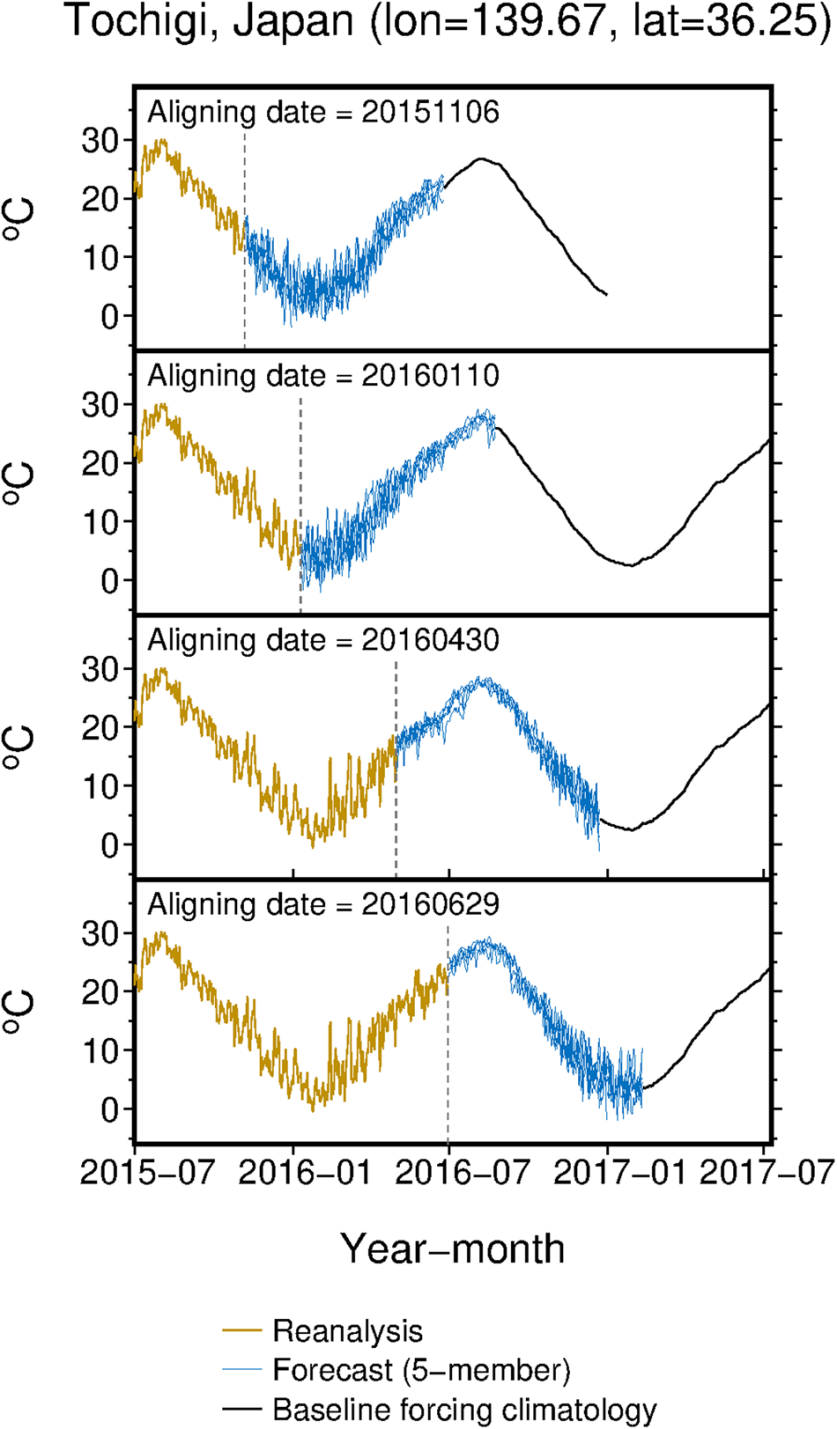
Example of JCDS 2-m air temperature data for different aligning dates. Data shown span the time period from July 1, 2015 to July 10, 2017, in a paddy-dominant area in Tochigi, Japan. The four aligning dates are presented: November 6, 2015 (20151106), January 10 (20160110), April 30 (20160430), and June 29, 2016 (20160629). Dashed vertical lines indicate aligning dates.

**Merging at aligning date**. Bias-corrected reanalysis and forecast data were integrated onto the baseline forcing climatology to produce a daily time series for three calendar years for each aligning date. The 3-yr period is centered on the year of the aligning date *t* and spans from January 1 of the previous year (*t*–1) to December 31 of the following year (*t*+1). Among the 24 initial conditions in the forecast, 241-day forecast data generated at the most recent date of issue before the aligning date were selected. Reanalysis data were then integrated into the series up to one day before the aligning date. This process was repeated for each forecast member, even though the reanalysis and baseline forcing climatology were the same, resulting in five different daily time series (Fig. 2). The proportion of reanalysis data in a given 3-yr period increased incrementally as the aligning date shifted to a point later in the year. Utilizing a 3-yr period allows for complete simulations of annual crop life cycles, i.e., from sowing to harvesting, irrespective of the timing of sowing and whether the crop duration spans two calendar years or is completed within a single year. Crop growth simulations driven by JCDS data can provide information for both monitoring (using the reanalysis portion) and forecasting (using the forecasting portion) (Fig. 1). Furthermore, crop growth simulations conducted using the JCDS data with different aligning dates can be used to derive yield predictions with different lead times, such as pre-sowing prediction, pre-harvest prediction, and post-harvest estimation, depending on the increase employed in the reanalysis portion of the JCDS data.

## Data Records

The JCDS dataset can be downloaded from the DIAS site for free with user registration^31^. The JCDS dataset offers global coverage, covering both terrestrial (including Antarctica) and ocean areas. The dataset spans 0° to 360° in longitude and −90° to 90° in latitude at a spatial resolution of 0.5° (~55 km around the equator) on a regular grid coordinate system. This corresponds to a matrix of 720 by 360 grid cells. At the time of writing, the JCDS dataset covers the period from 2010 to 2021 with respect to the aligning date *t*. Although somewhat irregularly spaced, the aligning dates are available for all months and are approximately 30 days apart (January 10, February 9, March 1, March 31, April 30, May 30, June 29, July 29, August 28, September 27, October 7, November 6, and December 6). Each aligning date directory within the dataset (e.g., “20100110” indicates that the date is January 10, 2010) contains 150 files, including five forecast members (labeled “e01” to “e05”), 10 variables, and data spanning a three-year period from *t*–1 to *t*+1, with *t* signifying the year of the aligning date. Each aligning date has a total data volume of approximately 35 GB, with files containing the daily data for that year. The total size of the aligning date data for the 12-yr period is approximately 416 GB. The repository is structured as JRA55_JMACPS2_Delta_S14FD/[aligning date]/[forecast ensemble]/[variable], where [aligning date] is for instance “20100110”; [forecast ensemble] is expressed like “e01”; and [variable] indicates climatic variable—“tave2m”, “tmax2m”, and “tmin2m” for daily mean, maximum and minimum 2-m air temperatures, respectively (°C); “precsfc” for precipitation rate (mm d^–1^); “dswrfsfc” and “dlwrfsfc” for downward shortwave and longwave radiation fluxes, respectively (W m^–2^); “rh2m” for relative humidity (%); “spfh2m” for specific humidity (kg kg^–1^); “wind10m” for 10-m wind speed (m s^–1^); and “pressfc” for surface pressure (hPa). For example, the directory “20100110/e01/tave2m” includes daily mean 2-m air temperature data for the 3-yr period from 2009 to 2011 (tave2m2009.nc4, tave2m2010.nc4, and tave2m2011.nc4) created on the aligning date of January 10, 2010 based on the first member of ensemble forecast. The file format is in NetCDF version 4.

## Technical Validation

For validation, the JCDS data were compared with actual climate data from the WFDE5, a bias-corrected version of ECMWF Reanalysis 5 that has been developed independently of the authors of this study^32^. A common statistical metric that is frequently used to quantify disparities between two datasets is the root mean square difference, *E*. According to Taylor^33^, *E* can be derived as the quadratic sum of the systematic component of the difference (or mean bias), $\bar{E}$, and the random component of the difference, ${E}^{{\prime} }$, as follows:

8 $${E}^{2}={\bar{E}}^{2}+{E}^{{\prime} 2}$$

The random component of difference for two datasets, *f* for JCDS and *r* for WFDE5, is defined by

9 $${E}^{{\prime} }=\sqrt{\frac{1}{N}\mathop{\sum }\limits_{n=1}^{N}{\left[\left(f-\bar{f}\right)-\left(r-\bar{r}\right)\right]}^{2}}$$

where $\bar{f}$ and $\bar{r}$ are the respective means, and *N* is the sample size, consisting of days and grid cells.

In this evaluation, it is plausible to attribute the bias primarily to the disparities in the different forcing datasets, i.e., S14FD, which is used as the baseline climatology in the JCDS dataset, and WFDE5, which is used as the reference dataset for the comparison. It is also reasonable to assume that the random component of difference comes mainly from errors in the forecast portion of the JCDS dataset. Consequently, the random component of difference is a more suitable metric than root mean square difference for assessing the accuracy of the JCDS in capturing actual climate variability, that is, once the biases in the JCDS dataset relative to the WFDE5 dataset have been discounted. We therefore used the random component of difference as the metric for quality assessment in the following subsections.

Comparisons for explanatory purposes were made for both a specific rice season at a single location in Japan, and for latitudinal zones encompassing global rice-producing areas. For the site-level assessment, the differences for the May-September rice-growing season were calculated for a single 0.5° grid cell situated in Tochigi Prefecture, Japan, where rice paddy cultivation is dominant (36.25°N; 139.67°E). The calculations were performed for each of seven aligning dates from April 30 to October 7. For the latitudinal zone-level assessment, calculations were performed for the summer rice season in the mid-latitudes of both the Northern Hemisphere (NH; May to September) and the Southern Hemisphere (SH; October to March), as well as the wet (July to December) and dry (January to May) rice seasons in the low-latitudes. These seasonal divisions were made based on the global rice calendar^34^.

### Site-level assessment

**Fig. 3:**
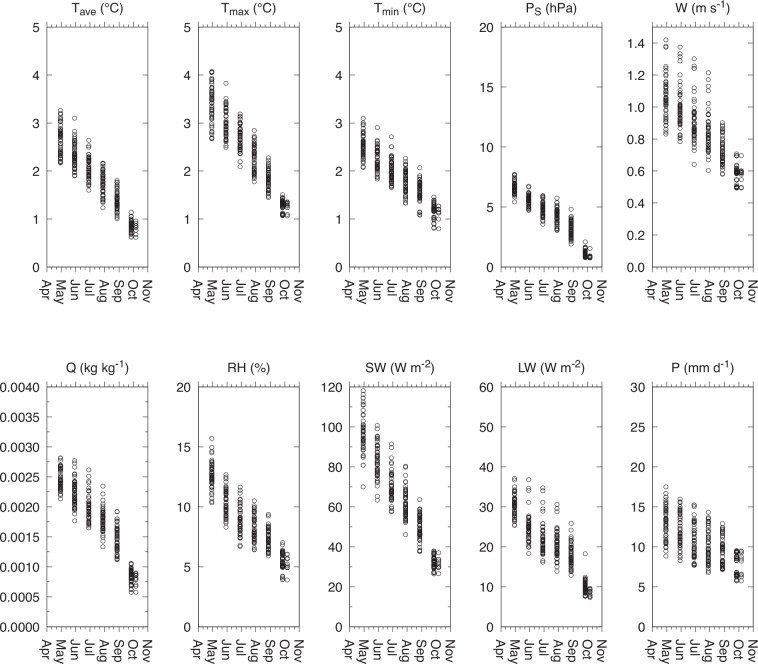
Changes in the quality of JCDS data at a specific location as a function of rice season progression. Data were calculated for a representative 0.5° grid cell located in Tochigi, Japan, where rice paddy cultivation is dominant (36.25°N; 139.67°E). The plots show the discrepancy between the JCDS and WFDE5 datasets across the rice-growing season (May to September) for aligning dates ranging from April 30 to October 7. For each aligning date, data for five members over a 10-yr period (2010–2019) are shown.

The results clearly showed that the discrepancies between the JCDS and WFDE5 datasets decreased as the rice season advanced, irrespective of the variables considered (Fig. 3). The proportion of the reanalysis (forecast) data in the JCDS dataset for the May-September period increased (decreased) over time. This suggests that the quality of the JCDS data for a fixed time period is a function of the aligning date. For the last aligning dates of October 7 in that rice season, the JCDS data are exclusively reanalysis data. Consequently, the discrepancies are mainly attributed to differences in reanalysis datasets between the JCDS data (which utilize JRA55) and the reference data (which utilize ERA5).

### Latitudinal zone-level assessment

**Fig. 4:**
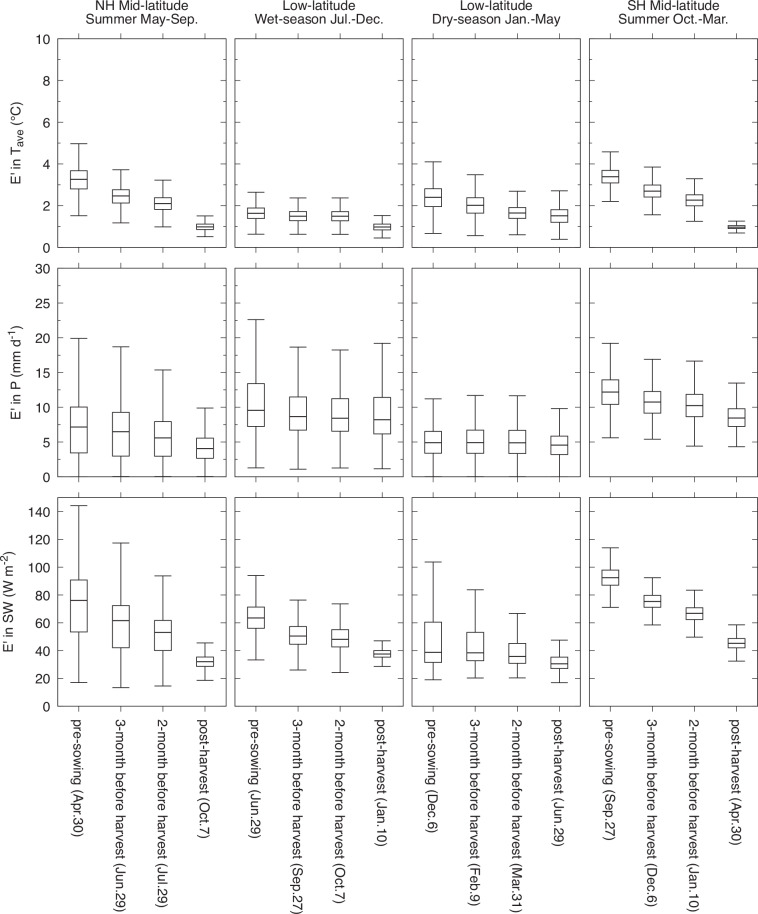
Evaluation of JCDS data quality for different latitudinal zones, seasons, and lead times until harvesting. The random component of difference (${E}^{{\prime} }$) calculated between the JCDS and WFDE5 datasets is used as the quality measure. Data for global rice-producing areas are presented in the mid-latitudes of the Northern Hemisphere (NH, poleward of 30°) and the Southern Hemisphere (SH, poleward of –30°), as well as the low-latitudes (equatorward of $\pm$30°). The mid-latitudes have a single summer rice season, whereas the low-latitudes have two rice seasons.

The findings at the latitudinal zone level corroborated the findings obtained for the site-level analysis. For the three variables considered, a decrease was observed in the disparity (or an increase in concordance) as the crop season progressed, both between zones and the two seasons in low-latitudes. On an average basis, the difference tended to be larger in absolute terms in the mid-latitudes of the NH and SH than in the low-latitudes, except for wet-season precipitation (Fig. 4). In the low-latitudes, smaller differences were observed in the wet season, on average, than in the dry-season for temperature, whereas the opposite trend was observed for precipitation and downward shortwave radiation flux. The results show that the maximal achievable limits of the quality of the JCDS dataset is modulated by regional and seasonal factors, primarily due to the variable skill of the climate forecasts employed.

### Limitations and future improvements

The current version of the JCDS can be improved. Recently, a new seasonal climate forecasting system—JMA/MRI-CPS3—has been released^35^ as a successor to JMA/MRI-CPS2. We plan to switch to JMA/MRI-CPS3 forecast data in future iterations of the JCDS. This integration will increase the number of initial dates from 24 to 73, and the number of ensemble members from five to 13, which will give users the option of using more frequent aligning dates as well as enhancing the quantification of forecast uncertainty. The aligning dates in the current version of the JCDS dataset are somewhat irregular due to initial development constraints imposed by differences in cropping seasons around the world. These aligning dates will be standardized in future iterations to facilitate usability. Nonetheless, even the current version of the JCDS dataset is a useful platform for developing and testing operational crop monitoring and forecasting services, as well as other applications.

Some of the variables in the forecast, such as *T*_max_, *T*_min_, *SW*, and *LW*, are not direct outputs of the climate model and were empirically estimated. More relevant climate model outputs will be used once they become available. Future work could also consider refining the spatial disaggregation procedure for forecast precipitation rate. Furthermore, alternative combinations of reanalysis, forecast, baseline forcing data, and bias-correction methods would be worth examining in future research. Finally, there is a need for comprehensive assessments of forecasting skill in predicting climate impacts (e.g., on crop yields) that integrate multiple climatic variables over specified timeframes, in addition to assessments of forecast skill of a particular climatic variable and seasonal climatic status^36,37^.

## Usage Notes

Here we present a case report using the JCDS dataset for predicting pre-sowing yield in the world’s major exporting country. For illustrative purposes, this case report focusses on the 2015/2016 dry-season rice production shortfall in Southeast Asia^38^. In Thailand, the world’s third largest rice exporter, the dry- or secondary season irrigated rice is typically planted in January and harvested in June. This harvest accounts for almost 30% of the annual production. The below-normal precipitation associated with the El Niño episode^39^ resulted in a dry-season rice yield that was almost two-thirds lower than the five-year average^40^.

**Fig. 5:**
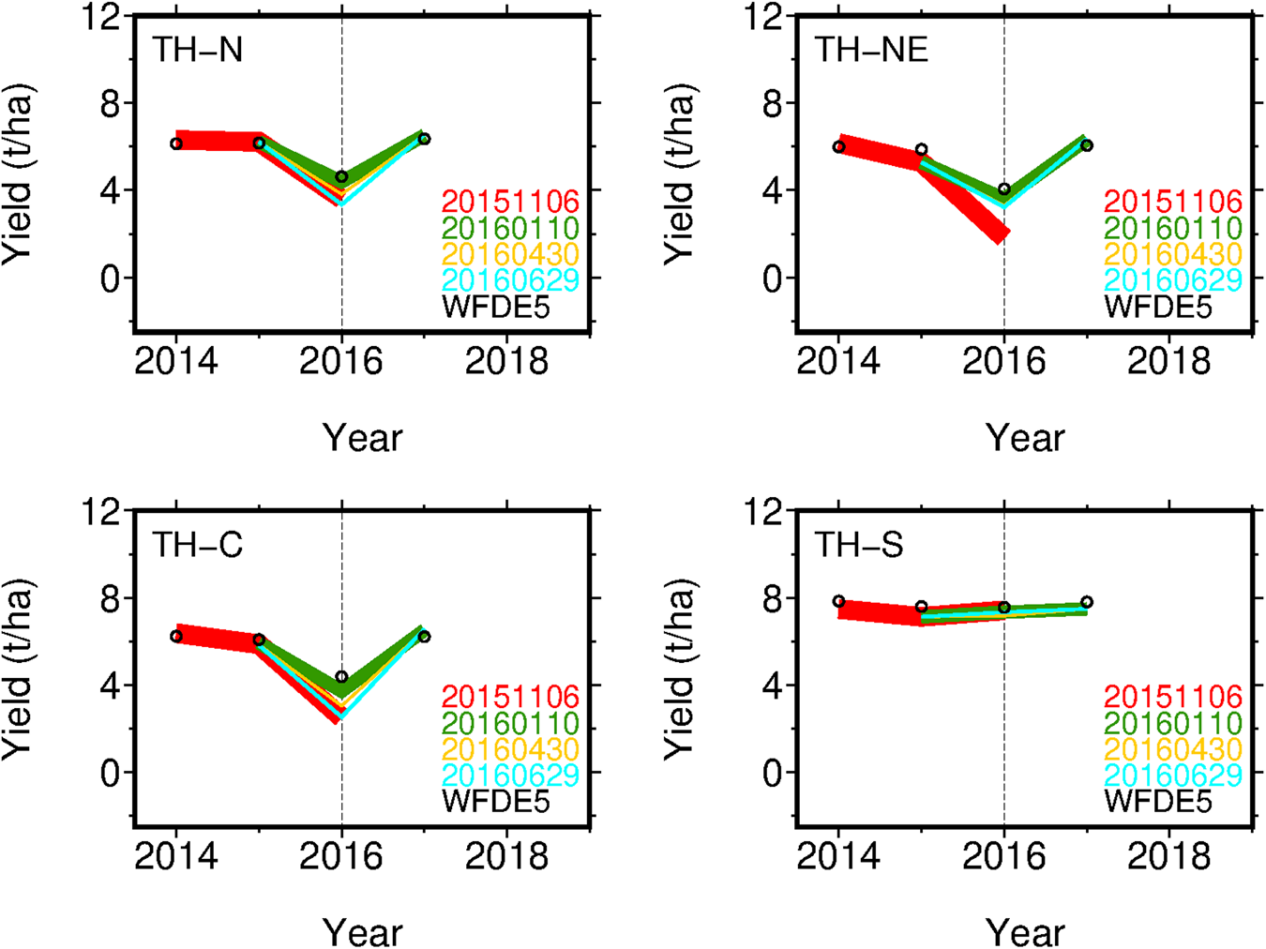
Forecasted regional average yields for the 2015/16 dry-season irrigated rice in Thailand (TH). N, NE, C, and S denote the northern, northeastern, central, and southern regions of that country, respectively. The forecasts were made using a single forecast member (e01) from the JCDS dataset with the aligning dates of November 6, 2015 (notated as 20151106) for the pre-sowing prediction, through January 10 and April 30, 2016 for the initial and subsequent pre-harvest predictions, respectively, to June 29, 2016 for the post-harvest estimate. WFDE5-based yields serve as benchmarks based on actual climate conditions.

**Fig. 6:**
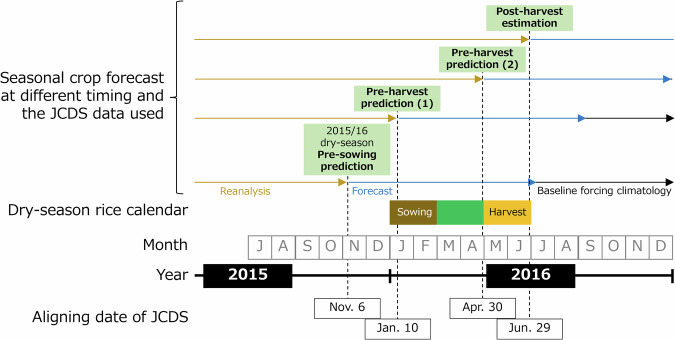
Schematic illustration of seasonal crop forecasting from pre-sowing prediction to pre-harvest prediction and post-harvest estimation. The 2015/16 dry-season rice in Thailand is taken as the example.

Using the CYGMA global gridded crop model that was previously validated for rice in Asian countries^41^, we verified that simulations forced by WFDE5 accurately captured the reduced yield in Thailand during the 2015/2016 dry-season, compared to the adjacent years of the same season. This substantiates the feasibility of accurate post-harvest estimation for that season. Moreover, pre-sowing yield predictions performed using the JCDS dataset accurately captured the decrease in yield as early as November 6, 2015, i.e., approximately two months prior to planting (Fig. 5). Subsequent predictions, updated in January and April 2016, were unchanged, thereby indicating the robust climate signal associated with this particular decrease in yield. The predicted decrease in yield was marked in the northern, northeastern, and central regions of Thailand, whereas that predicted in the southern region was as high as yields in adjacent years. The regional yield disparities were consistent across the pre-sowing (November 2015), pre-harvest (January and April 2016), and post-harvest (June 2016) estimates. Sequential provision of yield forecasts, from pre-sowing to mid-season to post-harvest (Fig. 6), can serve as an important tool for producers, agro-businesses, and national and international food agencies to preemptively prepare for predicted production shortfalls and potential disruptions in markets and international trade.

## Data Availability

The JCDS dataset described in this article was generated using a series of purpose-built programs written in Fortran90 on a MacOS PC. These programs utilize libraries to handle the NetCDF format. Due to the complexity and size of source data, which use several file formats and occupy approximately 7 TB, the programs are not directly distributed. A sample script to read and extract the JCDS data at give locations is available at: 10.5281/zenodo.12597021.

## References

[1] Iizumi, T. *et al*. Prediction of seasonal climate-induced variations in global food production. *Nat. Clim. Change* **3**, 904–908, 10.1038/nclimate1945 (2013). 10.1038/nclimate1945

[2] Rippey, B. R. The U.S. drought of 2012. *Weather Clim. Extremes* **10**, 57–64, 10.1016/j.wace.2015.10.004 (2015). 10.1016/j.wace.2015.10.004

[3] Ben-Ari, T. *et al*. Causes and implications of the unforeseen 2016 extreme yield loss in the breadbasket of France. *Nat. Commun.* **9**, 1627, 10.1038/s41467-018-04087-x (2018). 29691405 10.1038/s41467-018-04087-x PMC5915531

[4] Beillouin, D., Schauberger, B., Bastos, A., Ciais, P. & Makowski, D. Impact of extreme weather conditions on European crop production in 2018. *Phil. Trans. R. Soc. B* **375**, 20190510, 10.1098/rstb.2019.0510 (2020). 32892735 10.1098/rstb.2019.0510 PMC7485097

[5] White, R. H. *et al*. The unprecedented Pacific Northwest heatwave of June 2021. *Nat. Commun.* **14**, 727, 10.1038/s41467-023-36289-3 (2023). 36759624 10.1038/s41467-023-36289-3 PMC9910268

[6] Sawano, S. *et al*. Modeling the dependence of the crop calendar for rain-fed rice on precipitation in Northeast Thailand. *Paddy Water Environ.* **6**, 83–90, 10.1007/s10333-007-0102-x (2008). 10.1007/s10333-007-0102-x

[7] Hayashi, K., Llorca, L., Rustini, S., Setyanto, P. & Zaini, Z. Reducing vulnerability of rainfed agriculture through seasonal climate predictions: A case study on the rainfed rice production in Southeast Asia. *Agric. Syst.* **162**, 66–76, 10.1016/j.agsy.2018.01.007 (2018). 10.1016/j.agsy.2018.01.007

[8] Fujisawa, M. & Kobayashi, K. Climate change adaptation practices of apple growers in Nagano, Japan. *Mitig. Adapt. Strateg. Glob. Change* **16**, 865–877, 10.1007/s11027-011-9299-5 (2011). 10.1007/s11027-011-9299-5

[9] Morita, S., Wada, H. & Matsue, Y. Countermeasures for heat damage in rice grain quality under climate change. *Plant Prod. Sci.* **19**, 1–11, 10.1080/1343943X.2015.1128114 (2016). 10.1080/1343943X.2015.1128114

[10] Rose, D. C. *et al*. Decision support tools for agriculture: Towards effective design and delivery. *Agric. Syst.* **149**, 165–174, 10.1016/j.agsy.2016.09.009 (2016). 10.1016/j.agsy.2016.09.009

[11] Casaburi, L., Kremer, M. & Mullainathan, S. Harnessing ICT to increase agricultural production: evidence from Kenya. *PEDL Res. Papers* 1–26, https://www.poverty-action.org/publication/harnessing-ict-increase-agricultural-production-evidence-kenya#:~:text=Search-,Harnessing%20ICT%20to%20Increase%20Agricultural%20Production%3A%20Evidence%20From%20Kenya,control%20group%20with%20no%20messages (2019).

[12] IPCC. in *Climate Change 2014: Impacts, Adaptation, and Vulnerability. Part A* (eds. *et al*.) Summary for policymakers (Cambridge Univ. Press, 2014).

[13] Deser, C., Knutti, R., Solomon, S. & Phillips, A. S. Communication of the role of natural variability in future North American climate. *Nat. Clim. Change* **2**, 775–779, 10.1038/nclimate1562 (2012). 10.1038/nclimate1562

[14] Zachow, M., Nóia Júnior, RdeS. & Asseng, S. Seasonal climate models for national wheat yield forecasts in Brazil. *Agric. For. Meteorol.* **342**, 109753, 10.1016/j.agrformet.2023.109753 (2023). 10.1016/j.agrformet.2023.109753

[15] Ceglar, A. *et al*. Land-surface initialisation improves seasonal climate prediction skill for maize yield forecast. *Sci. Rep.* **8**, 1322, 10.1038/s41598-018-19586-6 (2018). 29358696 10.1038/s41598-018-19586-6 PMC5778075

[16] Iizumi, T., Shin, Y., Kim, W., Kim, M. & Choi, J. Global crop yield forecasting using seasonal climate information from a multi-model ensemble. *Clim. Serv.* **11**, 13–23, 10.1016/j.cliser.2018.06.003 (2018). 10.1016/j.cliser.2018.06.003

[17] Ubilava, D. The ENSO effect and asymmetries in wheat price dynamics. *World Dev.* **96**, 490–502, 10.1016/j.worlddev.2017.03.031 (2017). 10.1016/j.worlddev.2017.03.031

[18] Kunimitsu, Y. & Iizumi, T. Reproducibility of forecasting agricultural price fluctuations several months ahead of the harvest time. *Jpn. Agric. Res. Q.* **56**, 375–388, 10.6090/jarq.56.375 (2022). 10.6090/jarq.56.375

[19] van der Velde, M. & Nisini, L. Performance of the MARS-crop yield forecasting system for the European Union: Assessing accuracy, in-season, and year-to-year improvements from 1993 to 2015. *Agric. Syst.* **168**, 203–212, 10.1016/j.agsy.2018.06.009 (2019). 30774183 10.1016/j.agsy.2018.06.009 PMC6360854

[20] Ohno, H., Sasaki, K., Ohara, G. & Nakazono, K. Development of grid square air temperature and precipitation data compiled from observed, forecasted, and climatic normal data. *Clim. Biosphere* **16**, 71–79, 10.2480/cib.J-16-028 (2016). 10.2480/cib.J-16-028

[21] Kobayashi, S. *et al*. The JRA-55 Reanalysis: general specifications and basic characteristics. *J. Meteorol. Soc. Jpn.* **93**, 5–48, 10.2151/jmsj.2015-001 (2015). 10.2151/jmsj.2015-001

[22] Takaya, Y. *et al*. Japan Meteorological Agency/Meteorological Research Institute-Coupled Prediction System version 2 (JMA/MRI-CPS2): atmosphere–land–ocean–sea ice coupled prediction system for operational seasonal forecasting. *Clim. Dyn.* **50**, 751–765, 10.1007/s00382-017-3638-5 (2018). 10.1007/s00382-017-3638-5

[23] Iizumi, T., Takikawa, H., Hirabayashi, Y., Hanasaki, N. & Nishimori, M. Contributions of different bias-correction methods and reference meteorological forcing data sets to uncertainty in projected temperature and precipitation extremes. *J. Geophys. Res. Atmos.* **122**, 7800–7819, 10.1002/2017JD026613 (2017). 10.1002/2017JD026613

[24] Hay, L. E., Wilby, R. L. & Leavesley, G. H. A comparison of delta change and downscaled GCM scenarios for three mountainous basins in the United States. *J. Am. Water Resour. Assoc.* **36**, 387–397, 10.1111/j.1752-1688.2000.tb04276.x (2000). 10.1111/j.1752-1688.2000.tb04276.x

[25] Hawkins, E., Osborne, T. M., Ho, C. K., Challinor, A. J. Calibration and bias correction of climate projections for crop modelling: An idealised case study over Europe. *Agric. For. Meteorol*. 19–31. 10.1016/j.agrformet.2012.04.007 (2013).

[26] Iizumi, T. S14 global meteorological forcing dataset. *DIAS*10.20783/DIAS.523 (2017).

[27] Smagorinsky, J. in *Physics of Precipitation: Proceedings of the Cloud Physics Conference, Woods Hole, Massachusetts, June 3–5, 1959* (eds. Smith, W. E. & Weickmann, H.) On the dynamical prediction of large-scale condensation by numerical methods, 10.1029/GM005p0071 (1960).

[28] Ohno, H. & Isa, S. A statistical relation between GMS—viewed cloud amount and relative humidity. *Tenki* **31**, 493–495, https://www.metsoc.jp/tenki/pdf/1984/1984_08_0493.pdf (1984).

[29] Finch, J. W. & Best, M. J. The accuracy of downward short- and long-wave radiation at the earth’s surface calculated using simple models. *Meteorol. Appl.* **11**, 33–39, 10.1017/S1350482703001154 (2004). 10.1017/S1350482703001154

[30] Torralba, V., Doblas-Reyes, F. J. & Gonzalez-Reviriego, N. Uncertainty in recent near-surface wind speed trends: a global reanalysis intercomparison. *Environ. Res. Lett.* **12**, 114019, 10.1088/1748-9326/aa8a58 (2017). 10.1088/1748-9326/aa8a58

[31] Iizumi, T. JRA55-JMACPS2-Delta-S14FD reanalysis-forecast combined meteorological forcing dataset. *DIAS*10.20783/DIAS.649 (2022). 10.20783/DIAS.649

[32] Cucchi, M. *et al*. WFDE5: bias-adjusted ERA5 reanalysis data for impact studies. *Earth Syst. Sci. Data* **12**, 2097–2120, 10.5194/essd-12-2097-2020 (2020). 10.5194/essd-12-2097-2020

[33] Taylor, K. E. Summarizing multiple aspects of model performance in a single diagram. *J. Geophys. Res. Atmos.* **106**, 7183–7192, 10.1029/2000JD900719 (2001). 10.1029/2000JD900719

[34] Laborte, A. *et al*. RiceAtlas, a spatial database of global rice calendars and production. *Sci Data* **4**, 170074, 10.1038/sdata.2017.74 (2017). 28556827 10.1038/sdata.2017.74 PMC5448352

[35] Hirahara, S. *et al*. Japan Meteorological Agency/Meteorological Research Institute Coupled Prediction System Version 3 (JMA/MRI–CPS3). *J. Meteorol. Soc. Jpn.* **101**, 149–169, 10.2151/jmsj.2023-009 (2023). 10.2151/jmsj.2023-009

[36] Takaya, Y. *et al*. Skilful predictions of the Asian summer monsoon one year ahead. *Nat. Commun.* **12**, 2094, 10.1038/s41467-021-22299-6 (2021). 33828093 10.1038/s41467-021-22299-6 PMC8027800

[37] Masutomi, Y. *et al*. Systematic global evaluation of seasonal climate forecast skill for monthly precipitation of JMA/MRI-CPS2 compared with a statistical forecast system using climate indices. *J. Meteorol. Soc. Jpn.* **101**, 209–227, 10.2151/jmsj.2023-014 (2023). 10.2151/jmsj.2023-014

[38] Oxford Business Group. *Thailand’s drought weakens agricultural outlook*, https://oxfordbusinessgroup.com/news/thailand%E2%80%99s-drought-weakens-agricultural-outlook (2016)

[39] UNDP/OCHA/ESCAP/RIMES/APCC. *Enhancing resilience to extreme climate events: lessons from the 2015-2016 El Nino event in Asia and the Pacific*, https://repository.unescap.org/bitstream/handle/20.500.12870/551/ESCAP-2017-PB-El-Nino-Enhancing-resilience-to-extreme-climate-events.pdf?sequence=1&isAllowed=y (2017).

[40] FAO/GIEWS. *GIEWS Country Brief. Thailand*, https://www.fao.org/giews/countrybrief/country/THA/pdf_archive/THA_Archive.pdf (2023).

[41] Iizumi, T., Masaki, Y., Takimoto, T. & Masutomi, Y. Aligning the harvesting year in global gridded crop model simulations with that in census reports is pivotal to national-level model performance evaluations for rice. *Euro. J. Agron.* **130**, 126367, 10.1016/j.eja.2021.126367 (2021). 10.1016/j.eja.2021.126367

